# Using MoTR to Probe Agreement Processing in Russian

**DOI:** 10.1162/OPMI.a.35

**Published:** 2025-10-29

**Authors:** Metehan Oğuz, Cui Ding, Ethan Gotlieb Wilcox, Zuzanna Fuchs

**Affiliations:** Department of Linguistics, University of Southern California, Los Angeles, USA; Department of Computational Linguistics, University of Zürich, Zürich, Switzerland; Department of Linguistics, Georgetown University, Washington, DC, USA

**Keywords:** sentence processing, gender agreement, Russian, mouse tracking for reading, psycholinguistics

## Abstract

One important distinction in the syntax literature is between agreement that is *external* to the nominal phrase and agreement that is *internal* to it (sometimes called *concord*). How this type of agreement impacts sentence processing, however, is not well understood. In this paper, we ask whether agreement errors are processed differently based on their internal vs. external status. We investigate this question in Russian, a Slavic language that has a rich morphological agreement system and flexible word order, allowing us to control for several confounds. Our results are not fully conclusive but do provide moderate evidence that processing of agreement is modulated by internal vs. external status. We measure real-time language processing using Mouse Tracking for Reading (MoTR), a new web-deployable measurement tool that has been argued to improve over previous methods (e.g., self-paced reading) but has so far been tested only in English, and never for agreement processing phenomena. We find that MoTR can successfully pick up differences in our factorized psycholinguistic experiment in Russian, validating MoTR as a reliable tool for investigating agreement (error) processing. A direct comparison with existing data collected using in-lab eye-tracking-while-reading with similar experimental materials (Fuchs et al., 2025) suggests MoTR data yields larger effect sizes than does eye-tracking data.

## INTRODUCTION

### Background

A wealth of research has found that listeners and readers are sensitive to mismatches in grammatical agreement between a head noun and an associated linguistic element, such as an adjective, verb, or pronoun (Mancini, 2018; Pearlmutter et al., 1999). How the processing of agreement and agreement errors unfolds during real-time language processing has itself been a topic of major interest in the field, and it has also been used as a diagnostic in the investigation of the incremental processing of other phenomena, such as island sensitivity and binding constraints (e.g., Badecker & Straub, 2002).

It is generally agreed upon that not all agreement mismatches or errors are processed equally: research in this domain suggests that the processing of an agreement error in a particular feature^1^ may depend on the nature of the feature itself, including fine-grained properties that may not be overtly realized in the form of the morpheme encoding that feature (ex., defaultness). In the processing of number agreement, for instance, asymmetrical processing patterns have commonly been found in the processing of errors in singular vs. plural in English (e.g., Wagers et al., 2009), Spanish (e.g., Fuchs et al., 2015), and other languages (e.g., Deutsch & Dank, 2010). In languages with grammatical gender, like Spanish, asymmetrical patterns have also been found in the processing of agreement in masculine vs. feminine gender by monolingual and bilingual speakers (e.g., Beatty-Martínez & Dussias, 2019; Lago et al., 2015; Sá-Leite et al., 2019). This suggests that defaultness – the relationship of a feature’s possible values relative to each other – is among the properties of a feature that can modulate processing of agreement (errors) in real-time.

Still, in the work on the processing of agreement errors, an important distinction between internal and external agreement remains largely underexplored. In many languages with rich agreement systems, the elements that agree with features of the target noun can be divided into those that are internal to the nominal phrase, i.e., modifying adjectives, and those that are external to the target noun’s nominal phrase, i.e., verbs, predicative adjectives, pronouns, clitics, etc. The formal syntactic literature has investigated this matter, motivated by the observation that these subgroups of agreeing elements have different structural relationships to the head noun; in this literature, internal agreement is sometimes referred to as *concord* to distinguish it from external agreement. A substantial body of work in this vein suggests that internal and external agreement may underlyingly proceed from different mechanisms, and we point the reader to Chung (2013) and Norris (2014) for an overview of this debate in the formal syntactic literature. In the psycholinguistic literature, however, the lack of a difference in processing these two agreement types is often implicitly assumed. The question of whether there is evidence for a distinction between internal and external agreement during language processing remains open. Though exploratory, this question is motivated here by literature that suggests processing of agreement is modulated at least in part by the nature of the agreement feature itself. As discussed above, this vast literature makes clear that some properties of the mental representation of a word or its grammatical features (e.g., gender, number) that are not necessarily evident in the overt form may play a role in processing, and our aim is to determine whether the structural source of the feature (internal vs. external agreement) may be one of these properties.

There are a number of differences between internal and external agreement that may lead us to expect differential processing and resolution of errors in these two types of agreement. One consideration is the locality of the agreement relationship. Internal agreement is highly local, as it is restricted within the nominal phrase, whereas external agreement proceeds between the head of the nominal phrase and some external predicate. Depending on the language and the sentence, the linear and/or structural distance in the latter case can be quite large. Based on this alone, one might expect internal agreement to be easier to process and less effortful due to less taxation of memory, also leading to violations of this kind of agreement being penalized more heavily. On the other hand, external agreement is highly frequent, as in every clause there is a verb that has to index agreement with a head noun (though in *pro*-drop languages this noun may be phonologically null), whereas internal agreement is less frequent, as it occurs on adjectives, which are not obligatory. This might lead us to expect that agreement on adjectives could be more difficult to process. Indeed empirically there is evidence that children acquire agreement on adjectives later than other types of agreement (ex., in Spanish: Mariscal (2009)), and agreement within the nominal domain is more error-prone and vulnerable to attrition in certain bilingual populations (ex., Albirini et al. (2011); for Russian see Polinsky (2006)).

Only a few studies have tested this question, either directly or indirectly. Barber and Carreiras (2005) investigated the matter directly in an ERP study on Spanish, comparing the processing of agreement errors in determiner-noun pairs vs. adjective-noun pairs^2^. Results suggested differential negativity in these two types of agreement; however, this effect went away when the pairs (and thus the agreement manipulations) were embedded in a sentential context rather than presented in isolation. In a series of studies using the Visual World Paradigm, Hopp and Lemmerth (2018) and Lemmerth and Hopp (2019) found that monolingual speakers of German predictively processed gender agreement on determiners and adjectives preceding a noun, but that the effect was stronger when the agreeing element was an adjective than when it was a determiner. However, this finding was incidental to their research goals, and a possible confound relating to the nature of the material between the agreeing element and the noun presents some limitations in the interpretation of these findings as offering evidence in support of differential processing of external vs. internal agreement.

More recently, Fuchs et al. (2025) investigated the matter directly in studies on the processing of agreement errors using self-paced reading (SPR) and in-lab eye-tracking-while-reading. While many languages instantiate both internal and external agreement, this study targeted Russian; careful manipulation of certain properties of Russian controls for the confounds that can arise in a study on real-time processing of internal vs. external agreement, as will be discussed in the Internal vs. External Agreement in Russian section. Fuchs et al. (2025) predicted that if Russian speakers are sensitive to the type of agreement mechanism from which agreement arises on a particular agreeing element, then not only should participants show processing slow-downs in ungrammatical conditions – consistent with prior literature on processing of agreement errors – but that the strength of this effect should differ between elements that instantiate internal vs. external agreement. In both SPR and eye-tracking-while-reading, the authors found main effects of grammaticality, indicating that participants do indeed detect mismatches in agreement between an agreeing element and the target noun, but they did not find that the strength of this effect differed between internal and external agreement. However, this was a null finding, and it may have stemmed from certain limitations and trade-offs inherent to the employed methodologies. For the SPR task, while the sample size was larger due to the availability of participants online, the nature of the task is such that effects are often not localized, and the methodology does not allow regression to earlier text, which is an important element of naturalistic reading (Ferreira & Henderson, 1990; Jackson et al., 2012; Witzel et al., 2012). On the other hand, in-lab eye-tracking provides more localized measures of incremental processing, allows regressions, and is more naturalistic, but is also more resource-intensive, and the sample size for this task was expectedly smaller, potentially limiting power to detect an interaction effect.

In this study, we therefore aim to build on Fuchs et al. (2025) using a recently introduced methodology – Mouse Tracking for Reading (MoTR; Wilcox et al., 2024) – that combines the strengths of both of the previous methods (see Mouse Tracking for Reading section). In doing so, we can provide new insights into differential processing of internal vs. external agreement, using naturalistic reading-time data that is simultaneously not resource-intensive, as it is collected online and allows us to recruit a larger sample size at a lower cost. We therefore consider this an important opportunity to contribute evidence that MoTR can reliably detect effects of agreement errors – an empirical domain on which it has not been tested to-date (cf. Wilcox et al., 2024) – and that it can therefore be used in future work on the real-time processing of agreement (errors) and on phenomena for which reflexes of the processing of agreement errors serve as a diagnostic.

### Mouse Tracking for Reading

**Figure 1.**
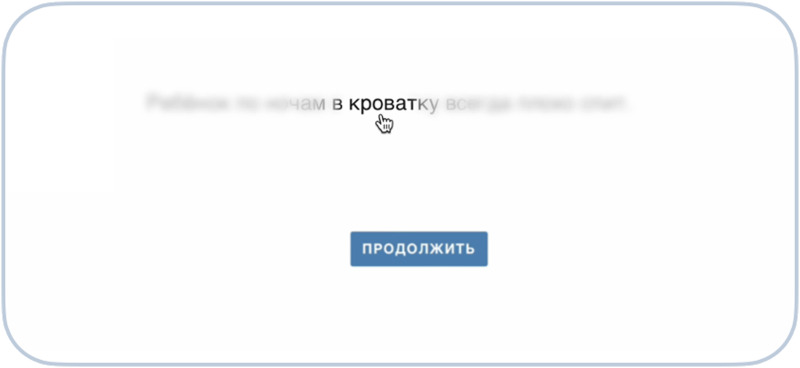
An example of a MoTR trial. The participant must move their mouse in order to reveal and read the text. Mouse movement is recorded and turned into a pattern of word associations, which reveal incremental processing times.

Mouse Tracking for Reading (MoTR) is a new experimental tool introduced in Wilcox et al. (2024) that can be used to collect by-word incremental processing times. In a MoTR trial, participants are presented with text on a computer screen. The screen is initially blurred, except for a small region around the tip of the mouse, referred to as the *spotlight*. In order to read the text, participants must move their cursor over the text that they wish to reveal. A visualization of a MoTR trial is presented in Figure 1. Cursor movement is recorded, producing a series of timestamped *x*/*y* coordinate positions at a sampling rate of 20 Hz. These coordinates can then be analyzed to extract incremental reading times (see below). The promise of MoTR is that it balances the cost of other web-deployable methods, such as self-paced reading, but allows for higher sensitivity and a greater range of experimental behaviors. In particular, it is possible to skip words and regress in MoTR, something that is not possible in other existing web-deployable methods, but which may be crucial for testing theories of language processing.

There are several parameters of a MoTR experiment that can impact the quality of the data collected. The first of these is the size of the spotlight. On the one hand, if the spotlight is too large (say, three or four words long) then the location of the mouse tip is potentially unaligned with the location of the participant’s gaze, or whichever word the participant is attending to. On the other hand, if the spotlight is too small, then the participant may be required to make many rapid movements, which could potentially be tiring or frustrating to the participant. In their original presentation of the method, Wilcox et al. (2024) use a spotlight size of 100 pixels (aligned with 12 pt font). Additionally, they set the spotlight band to be off-center relative to the tip of the mouse, to parallel the asymmetric nature of the human gaze (McConkie & Rayner, 1976; Rayner et al., 1980). Finally, the spotlight incorporates a gradual transition to blurriness around its edges in order to mimic the central focus and diminishing clarity of the human visual field (Rayner, 1998). This is evident in Figure 1, where the final -ky of kpobatky is blurred.

As stated above, a MoTR experiment produces a set of timestamped *x*/*y* coordinates. In order to turn these into incremental processing measures, following Wilcox et al. (2024), *attentional associations* (or *associations* for short) are computed. First, the word that the mouse tip was closest to at each time stamp is calculated; subsequently, all consecutive samples that occur across the same word are merged, giving the time that the word was closest to the center of the spotlight for that participant. These associations can be treated as something akin to fixation times in an eye-tracking experiment and can be used to compute parallels for well-used incremental processing measures, such as a word’s total reading time (the sum of all associations) or first pass gaze duration (the length of the association the first time the word is revealed).

How do participants use MoTR? Wilcox et al. (2024) show that there is a decent amount of individual variation, but that participants can be roughly shown to alternate between a few strategies. Sometimes they produce true fixations, where mouse forward movement is stopped, interspersing these with short, jerky mouse movements. This pattern of behavior mimics the fixation-saccade pattern observed in eye-tracking data. However, at other times participants produce more smooth scans over the text, speeding up at times and slowing down over words that are difficult to process, but never fully stopping their forward movement. Regardless of which pattern is used, the MoTR data presented in Wilcox et al. (2024) correlate well with human eye-tracking data and SPR data.

However, due to its novelty, MoTR remains a relatively untested experimental paradigm. Wilcox et al. (2024) ran factorized psycholinguistic tests in one domain, namely on interpretive preferences when reading sentences that contain attachment ambiguities. Furthermore, and perhaps more importantly, they only test the paradigm in English. The success of MoTR in these settings raises the possibility that it can serve as a low-cost, higher-precision experimental method; however, important questions remain about whether this tool can pick up effects for other psycholinguistic phenomena and languages that do not use the Latin alphabet.

### Internal vs. External Agreement in Russian

The present study uses MoTR to test the processing of internal vs. external agreement in Russian, a Slavic language that uses the Cyrillic alphabet and has three grammatical genders—masculine, feminine, and neuter. Agreement with the grammatical gender of a noun must be reflected in the form of several types of linguistic elements that might co-occur with the noun; most relevant to the present study are modifying adjectives (illustrated in (1)), predicative adjectives (2), and past tense verbs (1). That Russian marks gender agreement on all three of these categories makes it suitable for comparing the processing of agreement errors between internal agreement (modifying adjectives) and external agreement (predicative adjectives, verbs).

(1)  **modifying adjectives & verbs**
  a.  грязн**-ая** машина подъех-ал-**а** к дому
    dirty-f.sg.nom car.f.sg.nom arrive-pst-f.sg at house
    ‘(The) dirty car drove up to (the) house.’
  b.  грязн**-ый** трактор подъех-ал-∅ к дому
    dirty-m.sg.nom tractor.m.sg.nom arrive-pst-m.sg at house
    ‘(The) dirty tractor drove up to (the) house.’
(2)  **predicative adjectives**
  a.  машина всегда полез-**на**
    car.f.sg.nom always useful-f.sg.nom
    ‘(A) car (is) always useful.’
  b.  трактор всегда полез-**ен**
    tractor.m.sg.nom always useful-m.sg.nom
    ‘(A) tractor (is) always useful.’

There are two important possible confounds that constrain the languages in which the incremental processing of internal vs. external can feasibly be studied and that render Russian an ideal test case. The first consideration is linear order. A controlled study on internal vs. external agreement requires elements instantiating both types of agreement to occur in the same linear position relative to the head noun (e.g., immediately preceding it) across stimuli in different experimental conditions. This is difficult to achieve in languages with rigid word order. Russian has flexible word order: linear order of constituents can be manipulated such that any of the agreeing elements targeted here (modifying adjective, predicative adjective, verb) can occur before the target noun (3) and the sentence remains felicitous.

(3) 
  a.  грязн**-ая** машина…
    dirty-f.sg.nom car.f.sg.nom
    ‘(The) dirty car…’
  b.  подъех-ал-**а** машина к дому
    arrive-pst-f.sg car.f.sg.nom at house
    ‘(The) car drove up to (the) house.’
  c.  всегда полез-**на** машина
    always useful-f.sg.nom car.f.sg.nom
    ‘(A) car (is) always useful.’

A second possible confound in a study testing internal vs. external agreement is lexical category: the types of agreeing elements that instantiate internal vs. external agreement remain fixed in most cases (adjectives vs. verbs, respectively), and it is important to be able to identify the effects of processing different types of agreement over and above any differences in simply processing adjectives vs. verbs. Predicative adjectives (2) can disentangle these effects: they are of the same lexical category as modifying adjectives, but they are like verbs in that they instantiate external agreement. Crucially, Russian has different morphological marking on predicative vs. modifying adjectives, which allows the reader to disambiguate between the two types of adjectives and therefore the mechanism that yields agreement on that element. Modifying adjectives can only occur in the form of what is known in the literature on Russian as *long-form adjectives*, which take the agreement pattern illustrated in Table 1. Predicative adjectives can occur as either long-form adjectives or as what is known as *short-form adjectives* (Bailyn, 1994, 2012), which, as illustrated in Table 1, have distinct agreement marking from long-form adjectives. In the experimental paradigm discussed below, modifying adjectives occur only in the long form (consistent with their real-world use), and predicative adjectives only occur in the short form^3^. A comparison of processing patterns on these two types of adjectives allows us to disentangle effects of agreement type from effects of lexical category, per the discussion above.

**Table 1.** **Morphological forms:** Agreement suffixes for verbs and for long-form and short-form adjectives in Russian, with Cyrillic script and transliteration in Latin script below. In our experiments, the long form is constrained to appear on modifying adjectives only; the short form is used only for predicative adjectives, including outside of the context of the experiment.

	LF adjective	SF adjective	Verb
	*star-* ‘old’	*star-* ‘old’	*beža-* ‘run’
masculine	стар-ый	стар	бежал
	star-yj	star-∅	bežal-∅
feminine	стар-ая	стар-а	бежал-а
	star-aja	star-a	bežal-a

### Present Study & Research Questions

The present study uses MoTR to investigate the processing of agreement errors in Russian to address both theoretical and methodological research questions. From the theoretical perspective, we take the opportunity to further investigate whether processing of agreement (errors) is sensitive to the nature of the agreement mechanism instantiated by a given agreement target. Building on previous work, we compare agreement on modifying adjectives, verbs, and predicative adjectives in Russian, as this three-way comparison allows us to test for effects of agreement mechanism while controlling for effects of lexical category.

**Research Question 1:** Is processing of agreement errors modulated by the type of agreement (internal vs. external) instantiated by the agreeing element?

If agreement type indeed modulates the processing of agreement (errors), we expect predicative adjectives and verbs to pattern together to the exclusion of modifying adjectives. As to which of these agreement types is expected to show stronger effects, given that the considerations discussed in the Background section offer compelling arguments that this effect could go either way, here we refrain from making predictions regarding the directionality of this effect. Due to the possibility of any differences in processing of adjectives vs. verbs being driven by differences in processing of lexical category (as discussed in the previous section), we control for this by additionally testing whether lexical category modulates the processing of agreement errors. If it does, we expect modifying adjectives and predicative adjectives to pattern together to the exclusion of verbs.

We use materials from previous work with some modifications (Fuchs et al., 2025) (Methods section). By implementing the study in MoTR, we obtain data on incremental language processing from naturalistic reading, similar in nature to the in-lab eye-tracking-while-reading data collected in the previous work (Fuchs et al., 2025) but collected online with access to a larger sample size.

We additionally make a direct comparison between the results of the present MoTR study (Methods section) and the previous in-lab eye-tracking study (Fuchs et al., 2025) to determine whether MoTR can reliably detect effects of grammaticality in the processing of agreement and thus be used in future language processing studies—both those that directly investigate increased processing effort due to agreement errors and those that use reflexes of the processing of agreement errors as a diagnostic for other phenomena. Additionally, our results will contribute evidence for whether MoTR can be used in studies targeting languages that do not use the Latin alphabet and are morphologically rich (Liversedge et al., 2016; Parshina et al., 2021; Zdorova et al., 2023).

**Research Question 2:** Does MoTR reliably detect slowdowns in language processing driven by agreement mismatches in a factorized psycholinguistic test?

## METHODS

### Experimental Design and Materials

Participants’ task was to read sentences and to answer comprehension questions that followed some of those sentences. We used a 2 × 2 × 2 within-subjects design, manipulating *Grammaticality* (grammatical vs. ungrammatical), *Agreement Type* (internal vs. external), and *Lexical Category* (verb vs. adjective). This would lead to eight logically possible conditions; however, given that there are no verbs that instantiate internal agreement, this design in fact led to six conditions that could be tested in Russian (summarized in Table 2).

**Table 2.** **Summary of experimental conditions**.

Agreeing element	Lexical Category	Agreement Type	Grammaticality
Modifying Adjective	Adjective	Internal	Grammatical
	Adjective	Internal	Ungrammatical
Verb	Verb	External	Grammatical
	Verb	External	Ungrammatical
Predicative Adjective	Adjective	External	Grammatical
	Adjective	External	Ungrammatical

Stimuli were divided into six regions (schematized in Table 3). Each stimulus started with a prepositional phrase or a dative-marked NP in the first region, which was followed by the agreeing element in the second region (verb or adjective). The third region (target region) was an inanimate noun (masculine or feminine) that determined agreement on the preceding agreeing element in the second region. The fourth and fifth regions each had single words that varied depending on the clause type, and were used as spillover regions. No stimuli had commas or dashes, which could potentially affect the way items were read.

**Table 3.** **Schema of experimental stimuli:** The target region is Region 3.

Region 1	Region 2	Region 3	Region 4	Region 5
PP	Modifying Adj.	Noun (m/f)	Spillover 1	Spillover 2
	Agreeing Verb			
Ndat	Pred. Adj.			

There are two aspects of our materials that are important to note here: First, we only used *inanimate* head nouns in our study, diverging from Fuchs et al. (2025), who used both animate and inanimate head nouns. Considering that data was collected over the internet, we aimed to keep our study short so that the participants could stay focused on the task. In selecting a subset of the previously used stimuli, we decided to control for animacy, given considerations from theoretical syntax that semantic (animate) gender and abstract (inanimate) gender may be different features (Kramer, 2015) and experimental evidence that participants are more sensitive to errors in semantic gender than abstract gender (Vigliocco & Franck, 1999).

Second, while Russian has a three-way gender system (feminine, masculine, and neuter), we used only feminine and masculine items. We did this for several reasons. First, including neuter would add more complexity into the study, and we wanted to test our hypotheses in simpler settings, at least initially. Second, neuter nouns are fewer in number compared to feminine and masculine nouns (estimated between 13 percent of the Russian lexicon, cf. Akhutina et al. (2001) p. 296; Comrie et al. (1996) p. 109; and 16.7 percent, cf. Slioussar and Samoilova (2015)). Third, and possibly related to the previous point, is that there is empirical evidence to suggest that the processing of neuter agreement differs from the other two genders, which could be due to the aforementioned relatively low frequency of neuter nouns and thus of neuter agreement, or due to the fine-grained featural specification of neuter (Slioussar & Malko, 2016). Finally, we constrained ourselves to using (a subset of) the materials from Fuchs et al. (2025), who do not use neuter nouns. We acknowledge, however, that the exclusions of neuter and animate nouns are potential limitations of the present study, which may impact the generalizability of our conclusions.

24 stimuli sets were created (8 for each type of agreeing element). All target sentences were manipulated to occur with a masculine and a feminine head noun in the critical region. The contrast between masculine and feminine is neutralized in plural agreement forms of adjectives and verbs in Russian, so only singular head nouns were used in targets. Ungrammatical sentences were created by manipulating the agreement on the agreeing element to match or mismatch the grammatical gender on the target noun. The gender of the target noun was manipulated as a control factor. Below, we only present stimuli with a feminine head noun (машина ‘car/machine’) but similar sentences were created with a masculine noun as well.

(4)  a. **modifying adjective conditions, grammatical**

На дороге грязн-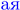 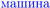 никак не может завестись.

on road dirty-f.sg.nom car.f.sg.nom nohow not able start

‘On (the) road, (the) dirty car cannot start.’

b. **modifying adjective conditions, ungrammatical**

*На дороге грязн-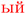 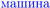 никак не может завестись.

on road dirty-m.sg.nom car.f.sg.nom nohow not able start

(5)  a. **verb conditions, grammatical**

K дому подъех-а-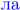 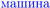 с выбитыми стеклами и фарами

to house arrive-pst-f.sg car.f.sg.nom with broken windows and headlights

‘A car with broken windows and headlights drove up to the house.’

b. **verb conditions, ungrammatical**

* K дому подъех-а-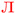 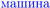 с выбитыми стеклами и фарами

to house arrive-pst-m.sg car.f.sg.nom with broken windows and headlights

(6)  a. **predicative adjective conditions, grammatical**

Дома всегда полезн-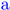 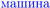 для заготовки фруктов

house always useful-f.sg.nom machine-f.sg.nom for preparation fruits 

и овощей

and vegetables

‘A machine for preparing fruits and vegetables is always useful at home.’

b. **predicative adjective conditions, ungrammatical**

* Дома всегда полезн-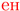 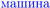 для заготовки фруктов

house always useful-m.sg.nom machine-f.sg.nom for preparation fruits

и овощей

and vegetables

Three experimental lists were created with 24 target items, 48 filler items and 4 practice items each and were assigned to participants randomly. Participants saw each item only in the grammatical or ungrammatical condition, so each participant saw 8 items per condition. Half of the fillers were grammatical, while the other half were ungrammatical due to violations in prepositional case forms. All items were maximally similar in length. In total, each participant read 72 experimental items. 42 of these items were followed by a yes/no comprehension question to ensure that participants read the sentences attentively.

### MoTR

The MoTR experiment was hosted on the GitHub page of one of the authors, and the front end was developed using the Magpie framework (https://magpie-ea.github.io/magpie-site), an open-source platform to create psychological experiments. Server infrastructure and database management were handled via Heroku (https://www.heroku.com/home), a cloud-based service that facilitates online data collection and management (Middleton & Schneeman, 2013).

We post-processed the raw MoTR data according to the pipeline in Wilcox et al. (2024), excluding fixations shorter than 160 ms or longer than 4000 ms. Various reading measures were calculated, with *gaze duration, go past time, total duration, first pass regression (FPReg)*, and *incoming regression (RegIn)* selected for analysis. Definitions of these measures are provided in Table 4. Gaze duration, go past time, and FPReg are first-pass reading measures, reflecting the cognitive processes that occur during the first pass through a sentence. Total duration and RegIn are non-first-pass measures, approximating re-evaluation or additional processing after the initial reading. Among these, gaze duration is considered an “early” measure, while go past time, total duration, FPReg and RegIn are “late” measures.

**Table 4.** **Reading measures:** These are computed from raw MoTR data following the procedure outline in Wilcox et al. (2024).

Type	Measure	Abbrev.	Description
Continuous	Gaze duration	None	Time durations spent fixating on a word during the first pass before moving forward or making a regression.
	Go past time	None	Cumulative duration of all fixations from the first encounter of a word until the reader progresses to a word to the right.
	Total duration	None	Sum of all fixations on a word, including first-pass and any additional fixations.
Binary	First-pass regression	FPReg	1 if a regression was initiated during the first-pass reading of the word, and 0 otherwise.
	Incoming regression	RegIn	1 if a regression lands on the word, i.e., the word was fixated again after the first-pass reading, otherwise 0.

### Participants

We recruited 64 native speakers of Russian (mean age = 35.17, sd = 10.20) over Prolific (https://www.prolific.co). All participants lived in Russia at least for the first 18 years of their lives. No participant had been living abroad for longer than three years at the time of data collection. All participants had high accuracy with comprehension questions (at least 80%).

### Procedure

Participants followed the study link from Prolific, and were asked to confirm that they met the requirements to participate (i.e., they were a native speaker of Russian, were older than 18 years old, and had not lived outside Russia for longer than 3 years). Participants were instructed that they were going to read some sentences in Russian by uncovering parts of them using their mouse, and that they were going to answer questions after some sentences. A short practice session with four items preceded the experimental session.

In each trial, the test sentence appeared on the screen and was completely blurred, which made it unreadable. The participant moved their mouse over the sentence to uncover the parts of the sentence (cf. Figure 1). At the end of each trial, participants had to click a button under the test sentence, which said продолжить (‘continue’). Upon clicking this button, the next trial began and the procedure was repeated. In trials with comprehension questions, the test item was replaced with the comprehension question and two options (да/нет, ‘yes/no’) appeared under the comprehension question, and the participant selected the correct answer by clicking on it. After making their selections, participants had to click the same ‘continue’ button. This button also served as a fixation point for the mouse before the next item began. Participants were not able to click the ‘continue’ button without reading at least some parts of the sentence or without selecting a response to the comprehension question.

### Data Analysis

We analyzed the MoTR data from the present study and reanalyzed the eye-tracking data from Fuchs et al. (2025), following the same procedure for both. Our primary focus was on the critical region, defined as the third region in each sentence, containing the head noun. In addition, we analyzed the second region (containing the agreement target, i.e., modifying adjective, verb or predicative adjective) and regions four and five to capture any spillover effects (referred to as *Spillover 1* and *Spillover 2* in the original study). Participants with comprehension accuracy below 0.8 were excluded, resulting in the exclusion of one participant from the eye-tracking data and none from the MoTR data. All analyses were performed in *R* (version 4.4.1; (R Core Team, 2024)). While our research question aligns with Fuchs et al. (2025), we made several methodological adjustments in the analysis, which we elaborate on below.

Our regression model included fixed effects for grammaticality (match vs. mismatch), gender (feminine vs. masculine), agreeing element (modifying adjective, predicative adjective, verb), and their interactions, including two-way interactions between grammaticality and agreeing element and the three-way interactions. We included gender as a control predictor because we were interested in its possible interaction with grammaticality and agreeing elements. We did not include agreement type and lexical category as separate factors; instead, they are implicitly modeled through custom contrast coding of the agreeing element factor (see Custom Contrast Coding section). Other control predictors in Fuchs et al. (2025), such as lexical frequency, word length, region lengths, and trial number, were excluded. To account for individual- and item-level variability, the random-effects structure included by-participant and by-item intercepts and slopes for within-item designed independent variables, with correlations allowed within each grouping factor. All data and code are available at https://osf.io/emvdw/.

#### Custom Contrast Coding.

For the categorical predictors in our regression models, we applied custom contrast coding instead of uniformly using sum contrasts as in Fuchs et al. (2025). This allowed us to construct contrast matrices tailored to our hypotheses, directly addressing our research questions (UCLA IDRE, 2011; Schad et al., 2020). The set of contrasts we investigated were as follows, with a label for each contrast written in *italics*: Grammaticality (*Gram*; mismatch vs. match conditions), agreement type (*AgrType*; predicative adjective & verb conditions vs. modifying adjective conditions), lexical category (*LexCat*; modifying adjective & predicative adjective conditions vs. verb conditions), the interaction between grammatically and agreement type (*GramxAgrType*), and the interaction between grammatically and lexical category (*GramxLexCat*). For a deeper discussion of why we chose these contrasts, see Appendix A. Next, for each of these contrasts, we constructed a hypothesis matrix by extracting the associated comparison weights for each. The contrast matrix was then derived using the generalized inverse of the hypothesis matrix. Contrasts were scaled so that the effect sizes in milliseconds or probability space can be directly interpreted as the differences between conditions, where a positive coefficient estimate indicates increased processing effort, seen in longer reading times or more frequent regressions (Brehm & Alday, 2022). An overview of the hypothesis matrix is provided in Appendix A Table A.1, and the summary of the contrast matrix is in Table A.2.

#### Bayesian Hierachical Model.

We implemented a set of Bayesian hierarchical models on the reading measures of interest in Stan (Carpenter et al., 2017) using Rstan (Stan Development Team, 2024), which integrates Stan with an R programming environment. Formally, they are defined as:

$$y_{ij}=g\left(\beta_{0}+b_{0i}+c_{0j}+\sum_{p=1}^{P}\left(\beta_{p}+b_{pi}+c_{pj}\right)x_{p}\right),$$ (1)

where *y*_*ij*_ is the measured response for participant *i* on item *j*. For our experiments looking at reading times, *y*_*ij*_ ∈ ℝ, whereas for FPReg and RegIn our response variable is binary—representing a probability, i.e., *y*_*ij*_ ∈ {0, 1}. *β*_0_ is the intercept, and *b*_0*i*_ and *c*_0*j*_ are random intercepts for participants and items, respectively. The predictor variable *x*_*p*_ (*p* = 1, …, *P*) denotes the custom contrast coded predictors listed in the Custom Contrast Coding section and Table A.2, with *b*_*pi*_ and *c*_*pj*_ as random slopes for each participant and item. Predictors designed as between-item variables, such as AgrType and LexCat, were excluded from the by-item random slope structure. The likelihood function *g*(⋅) depends on the dependent variable type: for continuous reading times, it is a Lognormal distribution with a family-specific scale parameter *δ*, equivalent to a Normal distribution on logged, scaled data; and for binary variables (FPReg and RegIn), it is a Bernoulli distribution with a logical inverse link. To facilitate effect size interpretation, we back-transformed effect estimates from log space to their original scale (milliseconds or probability) after model fitting.

We fit each model with four parallel chains, each running 4,000 iterations, of which 2,000 were warm-up iterations. Convergence was examined using the $\hat{R}$-statistic and visual inspection of the trace plots (Gelman et al., 2014). We applied mildly informative priors (Gelman et al., 2014), adapted from the prior distribution used in Jäger et al. (2020), based on guidance from Nicenboim et al. (2021), Chapter 6, for setting appropriate priors. Details of priors are provided in Table 5.

**Table 5.** **Overview of prior distributions:** *β*_0_ denotes the intercept, *β_p_* the predictors, and *b* and *c* the by-participant and by-item random effects. Ω is the correlation matrix within the variance-covariance structure for each random effects group. It follows a Lewandowski-Kurowicka-Joe (LKJ) distribution, and setting the parameter to 2 favors correlations around zero (Lewandowski et al., 2009).

**RTs (continuous)**	**FPReg and RegIn (binary)**
*β*_0_ ∼ Normal(6, 1)	*β*_0_ ∼ Normal(0, 1)
*β_p_* ∼ Normal(0, 0.1)	*β_p_* ∼ Normal(0, 1)
*sd*(*b*), *sd*(*c*) ∼ Exponential(2)	*sd*(*b*), *sd*(*c*) ∼ Exponential(2)
Ω ∼ LKJ(2)	Ω ∼ LKJ(2)
*σ* ∼ Exponential(2)	

#### Bayes Factors for Model Comparison.

Based on our research questions, we want to know whether agreement type or lexical category best predicts the human processing data. To do so, we use Bayes factors to compare two competing models: (1) the *GramxAgrType model*, which is similar to the hierarchical models described earlier but includes only the interaction between grammaticality and agreement type in the interaction term, and (2) a *GramxLexCat model*, which includes only the interaction between grammaticality and lexical type. While 95% credible intervals from Bayesian hierarchical models indicate the range within which 95% of the parameter estimates lie—suggesting whether predictors (in this case, interaction effects) are necessary to explain the data—they do not allow for direct model comparison (Rouder et al., 2018; Royall, 2017). Bayes factors quantify the relative evidence provided by the data for two competing models as the ratio of their marginal likelihoods, providing a direct measure of the relative support for one model over another (Nicenboim et al., 2021; Schad et al., 2023). We chose Bayes factor also because it aligns with our Bayesian framework, accounts for parameter uncertainty rather than reducing comparisons to point hypotheses (Schad et al., 2023), and facilitates the comparison of non-nested models, as is the case with the *GramxAgrType* and *GramxLexCat* models. For more details of this analysis, including our criteria for interpreting the Bayes factor results, see Appendix C.

## RESULTS

**Figure 2.**
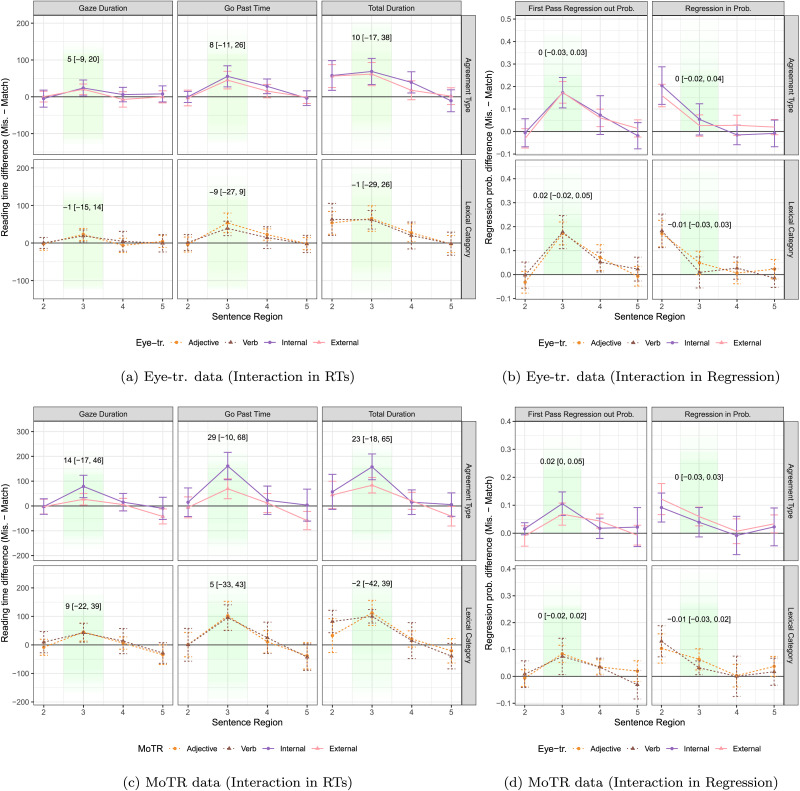
**Interactions in Eye-tr. and MoTR data.** (a) and (b) show interactions in Eye-tr. data; (c) and (d) show interactions in MoTR data. Top rows: Interaction between agreement type and grammaticality. Bottom rows: Interaction between lexical category and grammaticality. The green shaded area indicates the critical region. The line plots are for the raw reading measure data, which do not account for by-subject and by-item variability. Text in each grid displays the model-estimated back-transformed mean posterior of the interaction effect and its 95% credible interval. These values should roughly reflect the distance between the plotted lines (e.g., purple vs. pink solid lines or orange vs. brown dashed lines); note that, while the line plots do not account for random effects, the displayed values incorporate by-subject and by-item variability modeled in the Bayesian hierarchical analysis.

The results of our Bayesian analysis are presented in Table 6 (for eye-tracking) and Table 7 (for MoTR). Averages of the reading time data, broken into condition and sentence region, are presented in Figure 2. In the following subsections, we refer to these tables and figures as we walk through the results of each of our main research questions.

**Table 6.** **Bayesian analysis of eye-tracking data from Fuchs et al. (****2025****):** For reading measures at the critical region, each cell displays the posterior mean and 95% credible intervals of the effect sizes, back-transformed from log scale to milliseconds.

Effect (Eye-tr.)	Gaze Duration	Go Past Time	Total Duration	FPReg Prob.	RegIn Prob.
Gram	13 [1, 25]	38 [22, 55]	66 [41, 90]	.10 [.07, .14]	.03 [.01, .05]
Gen	−6 [−18, 7]	−5 [−20, 11]	0 [−25, 26]	.01 [−.01, .03]	.00 [−.02, .02]
AgrType	−7 [−31, 17]	−24 [−59, 10]	−42 [−97, 10]	−.02 [−.06, .02]	−.02 [−.04, .01
LexCat	−15 [−39, 10]	−45 [−81, −9]	−77 [−129, −23]	−.06 [−.09 −.02]	−.02 [−.04, .01
GramxAgrType	5 [−9, 20]	8 [−11, 26]	10 [−17, 38]	.00 [−.03, .03]	.00 [−.02, .04]
GramxLexCat	−1 [−15, 14]	−9 [−27, 9]	−1 [−29, 26]	.02 [−.02, .05]	−.01 [−.03, .03]
GramxGenxArgT	−5 [−18, 9]	0 [−15, 15]	6 [−18, 29]	.01 [−.01, .04]	−.01 [−.03, .01]
GramxGenxLexC	−4 [−18, 9]	−5 [−21, 11]	−11 [−34, 11]	.00 [−.03, .03]	.00 [−.02, .03]

**Table 7.** **Bayesian analysis of MoTR data:** For reading measures at the critical region, each cell displays the posterior mean and 95% credible intervals of the effect sizes, back-transformed from log scale to milliseconds.

Effect (MoTR)	Gaze Duration	Go Past Time	Total Duration	FPReg Prob.	RegIn Prob.
Gram	32 [6, 57]	72 [40, 105]	80 [45, 115]	.05 [.02, .07]	.02 [.00, .05]
Gen	−2 [−24, 20]	6 [−27, 39]	9 [−22, 39]	.01 [−.01, .02]	.01 [−.01, .03]
AgrType	−45 [−100, 11]	−67 [−126, -8]	−69 [−142, 7]	−.02 [−.03, .00]	−.02 [−.05, .02]
LexCat	−36 [−91, 18]	−51 [−108, 9]	−56 [−130, 18]	−.00 [−.02, .03]	−.01 [−.04, .04]
GramxAgrType	14 [−17, 46]	29 [−10, 68]	23 [−18, 65]	.02 [.00, .05]	.00 [−.03, .03]
GramxLexCat	9 [−22, 39]	5 [−33, 43]	−2 [−42, 39]	.00 [−.02, .02]	−.01 [−.03, .02]
GramxGenxArgT	15 [−9, 39]	22 [−9, 54]	33 [0, 66]	.00 [−.02, .02]	.01 [−.02, .03]
GramxGenxLexC	−2 [−28, 23]	−5 [−38, 29]	−13 [−47, 20]	.00 [-.01, .02]	.01 [−.03, .01]

### Does MoTR Detect Agreement Error Effects in Processing?

In their original study, Fuchs et al. (2025) observed grammaticality effects in the critical region across all reading measures except gaze duration. Our reanalysis of their eye-tracking data confirmed this, with sentences containing agreement mismatches requiring more processing effort, especially in late reading measures (Table 6, row 1). Mismatches led to 13 ms more processing time (CrI [1, 25] ms) for our early measure, gaze duration. In later measures, the differences were even larger—38 ms in go past time (CrI [22, 55] ms) and 66 ms in total duration (CrI [41, 90] ms). The agreement error effects are also shown in regression patterns: sentences with mismatches had a 0.10 higher probability of participants regressing to previous regions (CrI [0.07, 0.14]) and a 0.03 higher probability (CrI [0.01, 0.05]) of participants making regressions back into the critical region from subsequent regions.

MoTR data successfully replicated these grammaticality effects. As shown in Table 7 (row 1), mismatches in gaze duration led to an additional 32 ms processing time (CrI [6, 57] ms). For the go past time, the difference increased to 72 ms (CrI [40, 105] ms), and in total duration, mismatches added 80 ms (CrI [45, 115] ms). Regression patterns were consistent with the eye-tracking data as well: the probability of regressing to previous regions increased by 0.05 (CrI [0.02, 0.07]) in the mismatch condition, while regressions from subsequent regions back to the critical region increased by 0.02 (CrI [0.00, 0.05]).

### Is the Processing of Agreement Errors Modulated by Internal vs. External Agreement?

Our reanalysis of Fuchs et al. (2025) eye-tracking data found weak evidence for agreement type modulating the processing of agreement mismatch, while MoTR data provided somewhat stronger evidence for this modulation. The wide credible intervals in both data prevent us from drawing confident conclusions. To interpret these results, we consider three aspects: the sign of effects, effect sizes, and credible intervals, examining both main effects and interactions with grammaticality (including three-way interactions).

First, looking at the eye-tracking results, the AgrType predictor exhibits a negative effect across all reading measures (Table 6, row 3), indicating that nouns following external agreement elements (predicative adjective and verb) result in longer reading times compared to those following internal agreement elements (modifying adjective). This pattern is also reflected in increased regressions out of and into the nouns in the external agreement condition. Turning to the interaction term GramxAgrType (Table 6, row 5) in the eye-tracking data, we see a positive effect across all reading measures, suggesting that the mismatch penalty is larger in the internal agreement condition than in the external agreement condition. This interaction effect is also visualized in Figures 2(a) and (b), where the purple solid lines are slightly above the pink solid lines, reflecting the positive effect estimates by the model. The separation between them is very small, aligning with the wide credible intervals that include zeros.

The results from MoTR data lend stronger support to our hypotheses. First, we observed a larger negative main effect of AgrType across all reading measures, as shown in Table 7 row 3. This negative effect indicates that external agreement (predicative adjective and verb) causes longer reading times and more regressions on the nouns, compared to internal agreement (modifying adjective). Higher regression rates were also observed, with an increase of 0.02 in both FPReg and RegIn, though these credible intervals are wide and include zero. The interaction between agreement type and grammaticality is shown in Table 7 (row 5) and visualized in Figures 2(c) and (d). The interaction pattern in MoTR data aligns with eye-tracking data but is more pronounced. Internal agreement again shows a trend toward a larger mismatch penalty than external agreement, reflected in the greater distance between the solid purple and pink lines in the figure. Although the credible intervals remain wide, including zero, thus preventing us from drawing firm conclusions, the effect sizes in MoTR data are larger than those observed in eye-tracking. This makes zero as an effect size less probable in MoTR data, making the interaction pattern slightly clearer.

As a follow-up, we conducted a post-hoc analysis using total duration as a representative measure to compare mismatch effects within agreement types, focusing only on adjectives to control for lexical category. Using estimated marginal means via the *emmeans* package (Lenth, 2025), we examined modifying adjectives (internal agreement) and predicative adjectives (external agreement) separately. In the MoTR data, internal agreement showed a significant mismatch cost (40 ms, *p* = 0.0014), while the effect for external agreement was smaller and non-significant (13 ms, *p* = 0.29). In the eye-tracking data, both agreement types showed similar and significant mismatch costs (42 ms and 37 ms), respectively (both *p* < .0001). Our follow-up supports the pattern found in the main results: errors in internal agreement are harder for readers to process than errors in external agreement, at least in the MoTR data.^4^

### Is the Processing of Agreement Errors Modulated by Lexical Categories?

The results above showed a trend of crossover pattern in the GramxAgrType interaction—internal agreement is read faster than external agreement in the match condition, but about the same or slower in the mismatch condition, suggesting that the mismatch penalty is larger for internal than for external agreement, even though the interaction effect is small. However, we found no evidence of interaction between agreement error processing and lexical categories in either the reanalyzed eye-tracking data or MoTR data.

In the eye-tracking data, the main effect of lexical category is negative (Table 6 row 4), indicating that adjectives (modifying or predicative) lead to longer processing times than verbs on the critical region following them. Since most CrIs exclude zero, these results indicate a strong main effect of lexical category. However, the interaction between lexical category and grammaticality is negligible, with wide credible intervals centered around zero, making zero a very likely estimate. Unlike the GramxAgrType interaction, which reversed the sign of the main effect, the GramxLexCat interaction retains the same negative sign as the main effect. This lack of interaction is also visible in Figures 2(a) and (b), row 2, where the dashed orange lines for adjectives are slightly above or overlap with the brown verb lines.

The MoTR results reflect a similar pattern for the main effect of lexical category, with adjectives leading to longer processing times than verbs (Table 7, row 4). For regressions, the effect of lexical category is weaker, with mean estimates at 0 and −0.01 for regression out and in, and CrIs of [−0.02, 0.03] and [−0.04, 0.04], respectively. These intervals, all of which cross zero, suggest that the main effect of lexical category in MoTR data is less robust compared to the eye-tracking results. The interaction effect of GramxLexCat is shown in Figures 2(c) and (d) with dashed lines. The lines are almost entirely overlapping in the figure, suggesting that the processing of agreement (errors) is not modulated by lexical category. This interpretation is supported by the data in Table 7, row 6, where we find small estimates and wide CrIs centered around zero. These near-zero effect sizes and zero-centered CrIs suggest that agreement mismatch costs are likely similar for both adjectives and verbs, making a lexical category and grammaticality interaction unlikely in the MoTR data.

### Model Comparison Using Bayes Factors

**Figure 3.**
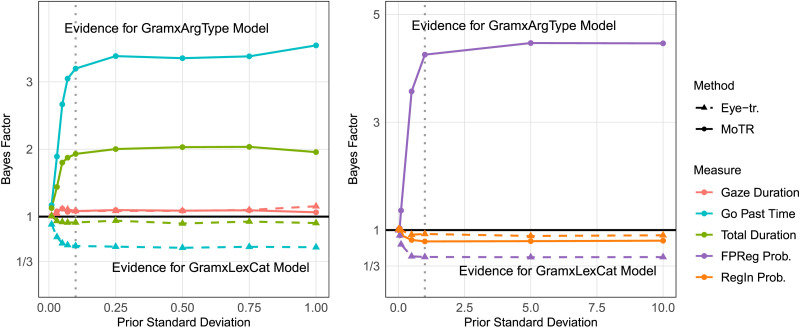
**Model comparison with Bayes factor and prior sensitivity checks:** The plots display the Bayes factor (*y*-axis) as a function of the prior standard deviation (*x*-axis) on GramxAgrType and GramxLexCat interaction effect for different measures and methods. The left panel focuses on reading time measures, while the right panel on binary regression measures. Solid lines represent results from the MoTR method, and dashed lines represent results from the eye-tracking method. Points indicate specific Bayes factor estimates. The vertical dotted lines indicate the prior *SD*s used in the Bayesian hierarchical models in the Bayesian Hierachical Model section. The horizontal line at *y* = 1 is the threshold between evidence favoring the GramxAgrType model (*y* > 1) and the GramxLexCat model (*y* < 1).

The Bayes factors for reading time measures (left panel in Figure 3) varied across methods and measures. Gaze duration provided only “anecdotal evidence” for the *GramxAgrType* model for both methods, with *BF* values near 1 regardless of prior standard deviation. In the go past time, MoTR data showed moderate evidence for the *GramxAgrType* model (*BF* > 3), particularly with larger prior *SD*s, whereas eye-tracking data weakly favored the *GramxLexCat* model (*BF* ≈ 0.6). Similarly, total duration in MoTR supported the *GramxAgrType* model with moderate evidence, while eye-tracking data showed weak anecdotal evidence for the GramxLexCat model (0.9 < *BF* < 1).

For the regression measures (right panel in Figure 3), first pass regression out probability (FPReg Prob.) provided moderate evidence favoring the GramxAgrType model in MoTR (*BF* > 4 for larger prior *SD*s), while eye-tracking data had anecdotal evidence against it (*BF* ≈ 0.5). Regression in probability (RegIn Prob.) consistently supported the *GramxLexCat* model across both methods, but the *BF* values remained close to 1 across all priors.

In summary, the Bayes factor model comparison shows that MoTR data moderately favored the GramxAgrType model while eye-tracking results were less conclusive.

## DISCUSSION

### Discussion of Research Question 1

Stepping back from the details, what evidence does the data lend to our hypothesis? Concerning the main results (i.e., not the Bayes factor analysis, which we will turn to in a moment), the main term we are interested in from our models is the interaction with grammaticality: Do we find an interaction between grammaticality and lexical category (*GramxLexCat*), or with agreement type (*GramxAgrType*)? To interpret our results, we will look at three properties of our models: First, is the coefficient for the interaction term consistent? Second, what is the magnitude of the effect? And third, what do the credible intervals look like?

First, it is very clear that the interaction between lexical category and grammaticality gives us consistently null results: The sign of the term is negative for eye-tracking data, but positive in two cases for MoTR data; the magnitude is very small (ranging in absolute value from 1–9 ms), and the confidence intervals are wide and centered at zero. However, agreement type gives us a different picture: The interaction terms *GramxArgType* are positive for all reading time metrics and measurement tools (i.e., both MoTR and eye-tracking). In addition, the effect size is larger, ranging from 5–28 ms. However, the credible intervals are still wide, meaning we cannot rule out the possibility of a zero effect.

These results suggest that agreement (error) processing is modulated by agreement type, conclusions which are also supported by our Bayes factor analysis. The analysis also suggests moderate evidence in favor of the *GramxArgType* model over the *GramxLexCat* model, at least for MoTR data when looking at go past times, total durations and FPReg probability. All evidence in favor of the *GramxLexCat* model was anecdotal (i.e., it had a Bayes factor of between 1 and 3).

**To sum up our findings**: We take these data as being consistent with the hypothesis that agreement processing difficulties are mediated by agreement type, and that internal agreement errors lead to more processing difficulties than external agreement errors. In line with findings in the formal syntactic literature that internal vs. external agreement may be driven by different underlying mechanisms (cf. Background section), this suggests that the distinction between internal and external agreement is also relevant for language processing studies. However, it is important to stress that the results are not strong enough to warrant a definitive conclusion, and, as discussed below, there are some unadressed limitations with our materials. We therefore conclude that further experiments are needed to definitively answer our first research question.

### Discussion of Research Question 2

The other main research question was whether MoTR could be used to pick up differences in controlled factorized psycholinguistic experiments in agreement phenomena and in languages that do not use the Latin alphabet. To answer this question, we ask whether the MoTR data shows clear and consistent effects that are in-line with the eye-tracking data. The results are strongly in the affirmative.

Looking at the main effects from our regression models, eye-tracking data shows strong positive effects and CrIs that do not overlap zero for *grammaticality* (i.e., there was a slowdown for ungrammatical sentences), as well as stronger effects for *agreement type* and *lexical category*. We find these same patterns replicated in the MoTR data—strong positive effects and CrIs that do not overlap zero for *grammaticality*, as well as stronger negative effects for *agreement type* and *lexical category*.

These results indicate that when significant effects from factorized psycholinguistics tests can be detected by eye-tracking methods, they can also be detected using MoTR. However, one might also want to know how the sensitivity of MoTR compares to the sensitivity of eye-tracking, in terms of its ability to detect significant effects. To answer this question, in Appendix D, we present two types of post-hoc power analyses comparing MoTR and eye-tracking for detecting a significant *GramxArgType* and *GramxLexCat* interaction, assuming these interactions exist. In the first experiment, we simulate 100 counterfactual versions of our experiment and ask, in what proportion do we find significant effects for each measurement tool? We find that MoTR has higher power, meaning it detects significant effects in a higher proportion of counterfactual datasets. In our second analysis, we simulate counterfactual datasets with different numbers of experimental stimuli and participants. Again, we find that MoTR is more likely to pick up significant effects than eye-tracking, but that a relatively high number of items (72) and participants (200) is needed to reach a power threshold of 0.8 (meaning significant interactions are found in 80% of the counterfactual datasets). We note, however, that, in general, more data is needed to observe interaction effects, which are more challenging to detect than main effects due to their smaller magnitude and greater susceptibility to noise. Overall, we take the results from these studies as further highlighting the potential cost benefits of MoTR: not only is MoTR cheaper to run on a per-trial basis than eye-tracking, but it also requires fewer participants and a smaller number of stimuli. For more details of this analysis see Appendix D.

One issue that these results raise is *why* we find larger effects in MoTR reading times compared to eye-tracking. One initial hypothesis is that eye-tracking while reading studies require participants only to control their eyes, while in MoTR they must coordinate hand movements. Therefore, participants are more likely to regress in eye-tracking, and an increased tendency to regress when encountering processing difficulty can result in shorter reading times on ungrammatical conditions. Indeed, we find that participants are about twice as likely to regress in ungrammatical conditions in eye-tracking, compared to MoTR. One other, related, possibility is that the manual nature of a MoTR trial forces participants to read the text more slowly, potentially causing them to pay attention to parts of the text that they may not notice if they were reading more quickly.^5^ This could also partially explain why larger effect sizes have been observed in other experimental measurement tools that involve more manual control, for example in the Maze task, where participants must choose between two options, which requires not only pressing the correct (of two) buttons with one finger, but also suppressing the pressing of the button corresponding to the incorrect continuation with the other finger (Boyce et al., 2020; Forster et al., 2009).

**In summary:** We conclude that MoTR can replicate the trends observed for eye-tracking in factorized psycholinguistic tests beyond languages with Latin alphabets, and in other phenomena that are commonly studied in psycholinguistics. In particular, our study investigated Russian, which, while still Indo-European, differs from English in several key ways that may affect reading-based measures of real-time processing, including having a different script, being more morphologically rich, and having flexible word order (Liversedge et al., 2016; Parshina et al., 2021; Zdorova et al., 2023).

### Limitations

The current study has several limitations, which are important to acknowledge. First, as mentioned in the Methods section, although Russian has a three-way gender system, with feminine, masculine, and neuter, the current study uses a subset of the target stimuli from Fuchs et al. (2025), and thus it is limited to gender mismatches of inanimate feminine and masculine nouns. We predict that our results should generalize to animate nouns and neuter nouns, though independent experimental evidence suggests effects could be weaker with neuter gender (Slioussar & Malko, 2016) or stronger for animate nouns (Vigliocco & Franck, 1999); we acknowledge that the generalizibility of our results to these types of nouns is an open question.

Second, although Russian is ideal in many respects for testing our research questions, certain properties of the language still introduce possible confounds or limitations. For instance, there is a length difference between modifying and predicative adjectives, with the former being (phonetically) one syllable and (orthographically) one letter longer than the latter. This presents a potential confound, as length could affect MoTR response times. Additionally, despite our experimental manipulation constraining the use of long-form agreement marking to modifying adjectives, it is nevertheless the case that in Russian outside of the context of the experiment, long-form adjectives are ambiguous between modifying and predicative adjectives (cf. Internal vs. External Agreement in Russian section); short-form adjectives are unambiguously predicative adjectives. Indeed, additional ambiguity may be further introduced by the fact that long-form and short-form adjectives have similar likelihood of occurring immediately preceding the head noun (based on the Russian National Corpus, cf. Fuchs et al. (2025)). In the present study, we assume that participants are fast learners in language experiments, and that the constrained use of this marking in our experimental design similarly reduces ambiguity in which type of agreement is being marked by the long-form adjective within the experiment, but we acknowledge this as a limitation. Future work may also consider languages with the same properties as highlighted for Russian in the Internal vs. External Agreement in Russian section, but with post-nominal modifying adjectives, which would allow for controlling of linear order among the three types of agreeing elements without resorting to non-canonical word orders (though see Fuchs et al. (2025) for discussion of frequency of the relevant constructions in the Russian National Corpus). One candidate language is Hebrew^6^, and we invite future work to engage with questions of internal vs. external concord in this language.

A third limitation of our study is the relatively small number of stimuli—8 for each type of agreeing element. One reason for this was that we wanted to match (a subset of) the stimuli from the original Fuchs et al. (2025) eye-tracking study. While our study did end up recruiting more participants than the eye-tracking study, we acknowledge that the small number of stimuli raises questions about the potential generalizability of our results.

Finally, the features (number and gender) in the non-critical positions in the stimuli were not systematically controlled. This might raise some questions regarding the possibility of interference or agreement attraction effects caused by the nouns that are irrelevant to our research questions. However, agreement attraction effects are typically the strongest when the attractor intervenes between the two elements that are in a dependency (as in *the keys to the cabinet are…). In our case, ancillary nouns do not intervene between the head noun and the agreeing element but instead precede both elements. This means that if interference effects were taking place, they would be introduced solely by the first noun of the sentence—a phenomenon known as *proactive interference*. While we do not expect such effects to be strong (evidence for proactive interference is inconsistent, and effects when found tend to be small, ex., Schoknecht et al. (2022)), we cannot rule them out as a potential confound. That being said, the random effect structure in our regression modeling should help to mitigate this variability.

## CONCLUSION

In this paper, we used a new incremental processing measurement tool to test the processing of gender agreement errors in Russian. As the main contribution, our results validate the use of MoTR for running factorized psycholinguistic tests (a) in a language other than English that crucially has a rich agreement system and uses a non-Latin alphabet, and (b) on agreement-processing phenomena. In addition, we found some evidence that processing is modulated by the type of agreement (internal vs. external), although the results are not conclusive.

Although further testing is needed on a variety of linguistic phenomena and languages with diverse properties to fully validate this MoTR as a tool, we take these results as an optimistic sign that MoTR can be used as a (cheaper) alternative to traditional eye-tracking methods for investigating agreement and other morphological phenomena in a variety of languages.

## ACKNOWLEDGMENT

We would like to thank Maria Polinsky, Olga Parshina, and Anna Runova for their help with the preperation of experiment materials. For helpful discussion, we would also like to thank the audience at the Marburg University Institute for German Linguistics, AMLaP 2024, CogSci 2024, and PsychoSlav 2024. Research presented in this paper was partially supported by Swiss National Science Foundation, grant 212276 (MeRID), PI: Lena Jäger. All errors are our own.

## Notes

^1^ The term “feature” is used here as short-hand for grammatical features such as phi-features, primarily number, grammatical gender, and person features.^2^ In some formal syntactic approaches to internal vs. external agreement, determiners are thought to instantiate external agreement, as their structural relationship to the noun (c-command) is similar to that of a verb rather than that of a nominal modifier like an adjective.^3^ We discuss possible limitations of this aspect of Russian and of the experimental design in the Limitations section.^4^ The effects observed in late MoTR and eye-tracking measures – such as go-past time, total duration, and regression rate – may reflect a deeper processing effort, specifically reanalysis, repair, or difficulty in integrating mismatched information, which may be particularly relevant to processing errors. As such, these results might suggest that the differences in external vs. internal agreement may unfold at the later stages of processing, i.e., during reanalysis and repair. As pointed out by an anonymous reviewer, the longer reading times and more regressions for errors observed for internal agreement as compared to external agreement might therefore suggest that repairs of errors in agreement between a target noun and the modifying adjective are more effortful, while the high frequency and/or increased structural distance in external agreement may allow readers to recover more easily from mismatches in this agreement type. This is indeed a promising direction; however, since our results are not strong enough to warrant a definitive conclusion, this discussion remains necessarily speculative.^5^ As pointed out by an anonymous reviewer, this assumption might predict that using MoTR in agreement attraction studies could potentially weaken attraction effects in illusory conditions, as spending more time on the attractor noun or the verb might cause these conditions to pattern more closely with ungrammatical conditions. This is an interesting prediction, and we invite future work to test this experimentally.^6^ We thank an anonymous reviewer for this suggestion.

## Figures and Tables

**Figure D.1.**
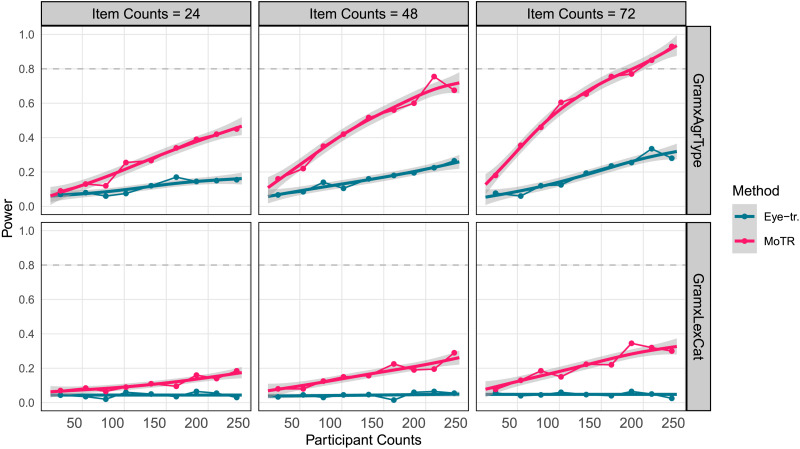
**Impact of varying participant and item counts on power across methods:** The Power of key interactions is plotted against participant counts, with separate panels for item counts (24, 48, and 72). Smoothed lines represent trends fitted using generalized additive models (GAM; Hastie (2017); Wood (2017)), with grey shaded areas indicating 95% confidence intervals around these trends. Dashed grey horizontal lines mark the power threshold of 0.8. Points and solid lines represent observed mean power estimates for each method, with MoTR in pink and eye-tracking in blue.

## APPENDIX A. DETAILS OF CONTRAST CODING

In this appendix, we present more information about the details of our contrast coding scheme. First, we rationalize the choices we made for our contrasts. Each of our contrasts was associated with a particular sub-question of our main, overall research questions, detailed in the Present Study & Research Questions section. Below, we outline each of these sub-questions and the contrast associated with each.

- **Do agreement errors increase processing effort?** We assessed grammaticality effects by comparing mismatch with match conditions, using a scaled sum contrast. This main effect, denoted as Gram, captures the overall processing effort caused by agreement errors.
- **Does the type of agreement instantiated by the agreeing element affect processing effort?** Agreement type effects were examined by contrasting internal agreement (modifying adjective) with external agreement (predicative adjective and verb). This main effect is denoted as AgrType.
- **Does the lexical category of the agreeing element affects processing effort?** We assessed lexical category effects by contrasting verbs (verb) with adjectives (modifying adjective and predicative adjective). This main effect is denoted as LexCat.
- **Does the effect of agreement errors vary by agreement type?** To investigate whether processing differences between agreement match and mismatch depend on agreement type, we examined the interaction between Gram and AgrType, denoted as GramxAgrType.
- **Does the effect of agreement errors vary by lexical category?** We assessed whether processing differences between agreement match and mismatch depend on the lexical category of the agreeing element by analyzing the interaction between Gram and LexCat, denoted as GramxLexCat.

Next, in Table A.1 and Table A.2 we present the hypothesis matrix and the contrast matrix, respectively, that are derived from these contrasts.

**Table A.1.** **Overview of hypothesis matrix:** Weights assigned to the categorical predictors and the interaction terms across experimental conditions in the regression models. They are derived from the sub-questions in Custom Contrast Coding and are scaled to reflect the contribution of each condition to the overall effect. The second section lists fixed-effect variables, and the third and fourth sections list two-way and three-way interaction terms.

	**Experimental condition**											
	**Masculine**						**Feminine**					
	**Mismatch**			**Match**			**Mismatch**			**Match**		
	**m-adj**	**verb**	**p-adj**	**m-adj**	**verb**	**p-adj**	**m-adj**	**verb**	**p-adj**	**m-adj**	**verb**	**p-adj**
Gram	1/6	1/6	1/6	−1/6	−1/6	−1/6	1/6	1/6	1/6	−1/6	−1/6	−1/6
Gen	1/6	1/6	1/6	1/6	1/6	1/6	−1/6	−1/6	−1/6	−1/6	−1/6	−1/6
AgrType	1/4	−1/8	−1/8	1/4	−1/8	−1/8	1/4	−1/8	−1/8	1/4	−1/8	−1/8
LexCat	−1/8	1/4	−1/8	−1/8	1/4	−1/8	−1/8	1/4	−1/8	−1/8	1/4	−1/8
GramxAgrType	1/4	−1/8	−1/8	−1/4	1/8	1/8	1/4	−1/8	−1/8	−1/4	−1/8	−1/8
GramxLexCat	−1/8	1/4	−1/8	1/8	−1/4	1/8	−1/8	1/4	−1/8	−1/8	−1/4	−1/8
GramxGenxArgT	1/2	−1/4	−1/4	−1/2	1/4	1/4	−1/2	1/4	1/4	1/2	−1/4	−1/4
GramxGenxLexC	−1/4	1/2	−1/4	1/4	−1/2	1/4	1/4	−1/2	1/4	−1/4	1/2	−1/4

**Table A.2.** **Overview of contrasts matrix:** Contrast coding of predictors for the models fit on MoTR data and eye-tracking data from Fuchs et al. (2025). The second section lists fixed-effect variables, and the third and fourth sections list two-way and three-way interaction terms.

	**Experimental condition**											
	**Masculine**						**Feminine**					
	**Mismatch**			**Match**			**Mismatch**			**Match**		
	**m-adj**	**verb**	**p-adj**	**m-adj**	**verb**	**p-adj**	**m-adj**	**verb**	**p-adj**	**m-adj**	**verb**	**p-adj**
Gram	1/2	1/2	1/2	−1/2	−1/2	−1/2	1/2	1/2	1/2	−1/2	−1/2	−1/2
Gen	1/2	1/2	1/2	1/2	1/2	1/2	−1/2	−1/2	−1/2	−1/2	−1/2	−1/2
AgrType	2/3	0	−2/3	2/3	0	−2/3	2/3	0	−2/3	2/3	0	−2/3
LexCat	0	2/3	−2/3	0	−2/3	−2/3	0	2/3	−2/3	0	2/3	−2/3
GramxAgrType	2/3	0	−2/3	−2/3	0	−2/3	2/3	0	−2/3	−2/3	0	2/3
GramxLexCat	0	2/3	−2/3	0	−2/3	−2/3	0	2/3	−2/3	0	−2/3	2/3
GramxGenxArgT	1/3	0	−1/3	−1/3	0	1/3	−1/3	0	1/3	1/3	0	−1/3
GramxGenxLexC	0	1/3	−1/3	0	−1/3	1/3	0	−1/3	1/3	0	1/3	−1/3

## APPENDIX B. COMPREHENSION QUESTION ACCURACIES

To provide an overview of the quality of online data collected via MoTR and in-lab data collected with eye-tracking, we calculated mean response accuracy for trials followed by comprehension questions across each experimental condition (Table B.1). Overall, MoTR accuracy rates were slightly higher than those from eye-tracking for most conditions. The mean accuracy levels are consistent across conditions as well, indicating comparable data quality and experimental design.

**Table B.1.** Mean comprehension question response accuracy for each experimental condition.

	**Accuracy by experimental conditions (%)**											
	**Masculine**						**Femine**					
	**Mismatch**			**Match**			**Mismatch**			**Match**		
	**m-adj**	**verb**	**p-adj**	**m-adj**	**verb**	**p-adj**	**m-adj**	**verb**	**p-adj**	**m-adj**	**verb**	**p-adj**
Eye-Tr.	91	96	93	93	97	93	95	97	95	86	94	92
MoTR	96	99	95	98	99	100	94	100	97	93	100	97

## APPENDIX C. BAYES FACTOR ANALYSIS DETAILS

This appendix provides additional technical details about our Bayes Factor analysis.

The analysis was conducted on all five reading measures, using data from both MoTR and eye-tracking methods at the critical sentence region. Given the sensitivity of Bayes factors to model specifications, particularly prior assumptions on the target effects, we conducted a Bayes factor sensitivity analysis by varying prior standard deviations for the interaction effects while keeping priors for other predictors constant (Schad et al., 2023; Schoknecht et al., 2025). For reading time measures, prior standard deviations ranged from 0.01 to 1 on the log scale, and for regression measures, from 0.01 to 10 on the logit scale. These ranges were translated into milliseconds for reading time measures and probabilities for regression measures (Table C.1).

**Table C.1.** **Overview of priors used for Bayes factor sensitivity analysis:** We vary the standard deviation of the prior for the interaction effects, and we also present their corresponding ranges in milliseconds for reading time measures and probabilities for regression measures.

**Reading time measures**		**Regression measures**	
**Prior**	**Range in ms**	**Prior**	**Range in prob.**
Normal(0, .01)	[−8, 8]	Normal(0, .01)	[−.005, .005]
Normal(0, .025)	[−19, 20	Normal(0, .05)	[−.024, .024]
Normal(0,. 05)	[−38, 42]	Normal(0, .1)	[−.05, .05]
Normal(0, .075)	[−55, 64]	Normal(0, .5)	[−.23, .23]
Normal(0, .1)	[−72, 87]	Normal(0, .1)	[−.38, .38]
Normal(0, .25)	[−156, 255]	Normal(0, .5)	[−.499, .499]
Normal(0, .5)	[−252, 671]	Normal(0, .10)	[−.5, .5]
Normal(0, .75)	[−311, 1351]		
Normal(0,. 1)	[−347, 2461]		

We followed the workflow suggested in Schad et al. (2023) to fit GramxAgrType and GramxLexCat models for each combination of method and reading measure. We estimate the marginal likelihood for each model using bridge sampling (Gronau et al., 2017), as implemented in the *R* package *bridgesampling* (Gronau et al., 2020). To ensure stable results, we used four chains in brms (Bürkner, 2021) with 20,000 iterations per chain, including 2,000 warm-up iterations, as recommended by Schad et al. (2023). Table C.2 provides a qualitative interpretation of Bayes factors based on Lee and Wagenmakers (2014), though these guidelines should not be treated as strict thresholds (Nicenboim et al., 2021).

**Table C.2.** **Qualitative descriptions of Bayes factors:** BF12 $=\frac{P\left(\text{data}|\text{model}\;1\right)}{P\left(\text{data}|\text{model}\;2\right)}$. A value greater than 1 indicates stronger support for model 1, while a value between 0 and 1 indicates stronger support for model 2. Larger deviations from 1 provide stronger support for one model over the other.

**Bayes Factor (BF**_**12**_ **)**	**Interpretation**
> 100	Extreme evidence
30–100	Very strong evidence
10–30	Strong evidence
3–10	Moderate evidence
1–3	Anecdotal evidence
1	No evidence

## APPENDIX D. POST HOC POWER ANALYSIS

To better understand the discrepancies between results from the MoTR and eye-tracking methods and to gain insights into how differences in statistical power might contribute to the divergence in findings between the two methods, we conducted a power analysis on reading time measures. This analysis aimed to evaluate the sensitivity of the experimental setup for each method in detecting the key interaction effects, namely the interaction between grammaticality and agreement type (GramxAgrType) and the interaction between grammaticality and lexical type (GramxLexCat), assuming these effects are present.

First, we simulated 100 datasets for the actual experimental setups in MoTR and eye-tracking experiments using the *designr* package (Rabe et al., 2023) Simulations were based on parameter estimates extracted from the Bayesian hierarchical models described in Bayesian Hierachical Model section, including fixed effects, random effect variances for participants and items, residual variances, and correlations among random effects. Data was generated using a linear mixed effects model that incorporated the above-extracted parameters. For each simulated dataset, we estimated the GramxAgrType and GramxLexCat interaction effects using another linear mixed effects model. To address the singularity issues, we excluded correlations among random effects and retained only random intercepts and random slopes for grammaticality. Power was calculated as the proportion of simulations where the interaction effect achieved statistical significance (*P* < 0.05). The results are summarized in Table D.1.

**Table D.1.** **Power estimates for actual experimental setup in MoTR and eye-tracking:** Estimates were for the key interactions across Gaze Duration, Go Past Time, and Total Duration. The number of participants(*P*) and trials(*T*) is indicated in parentheses for each method. GramxAgrType refers to the interaction between grammaticality and agreement type, while GramxLexCat refers to the interaction between grammaticality and lexical type.

Power Estimates	Gaze Duration	Go Past Time	Total Duration
**Eye-Tr.** (*P* = 39, *T* = 48)			
GramxAgrType	0.04	0.06	0.07
GramxLexCat	0.06	0.08	0.05
**MoTR** (*P* = 64, *T* = 24)			
GramxAgrType	0.18	0.33	0.18
GramxLexCat	0.10	0.11	0.02

For the eye-tracking method (*P* = 39, *T* = 48), power to detect the GramxAgrType interaction was consistently low across all measures (0.04−0.07), as was power for the GramxLexCat interaction (0.05 −0.08). In contrast, MoTR (*P* = 64, *T* = 24) showed higher power for GramxAgrType, particularly for go past time (0.33), while power for GramxLexCat remained low across measures (0.02−0.11).

These results indicate that MoTR, with its experimental setup, is more sensitive in capturing GramxAgrType interactions, especially for go past time. Both methods show limited sensitivity to GramxLexCat interactions.

Second, we explored how power estimates for GramxAgrType and GramxLexCat interactions in eye-tracking and MoTR data depend on participant and item counts. Using gaze duration as an example, we simulated 100 datasets for each combination of participant counts (ranging from 12 to 240) and item counts (from 24 to 72) following the earlier procedures. Power was calculated as before, and Figure D.1 shows how sensitivity to detecting these interactions varies with participant and item counts for both methods.

For GramxAgrType, the MoTR method showed a consistent increase in power with higher participant counts, particularly when item counts were also large. MoTR power reached 0.8 when item counts reaching 72 and participant counts exceeding 200. In contrast, the eye-tracking method displayed much lower sensitivity to GramxAgrType, with power rarely exceeding 0.4, even with 240 participants and 72 item counts. For GramxLexCat, the power remained generally low across both methods, with neither method achieving power values near the recommended threshold of 0.8. While MoTR had slightly better performance, reaching power values around 0.35 in scenarios with 72 items and more than 200 participants, the eye-tracking method consistently failed to exceed the power of 0.2 in capturing GramxLexCat interaction.

Interaction effects are generally more challenging to detect than main effects due to their smaller magnitude and greater susceptibility to noise, resulting in higher variance relative to their effect sizes and reduced statistical power. MoTR’s higher sensitivity to interactions may be attributed to its amplification of effects, as the physical effort required for hand or mouse movements is greater than that for eye movements.
